# The model of palliative care in the perinatal setting: a review of the literature

**DOI:** 10.1186/1471-2431-12-25

**Published:** 2012-03-12

**Authors:** Albert Balaguer, Ana Martín-Ancel, Darío Ortigoza-Escobar, Joaquín Escribano, Josep Argemi

**Affiliations:** 1 Department of Pediatrics, Hospital General de Catalunya, Universitat Internacional de Catalunya, Josep Trueta, s/n, 08195 Sant Cugat del Vallès (Barcelona), Spain; 2 Centre de Recerca i Estudis Bioètics. Institut d'Estudis Superiors de Bioètica. Universitat Internacional de Catalunya, Barcelona, Spain; 3 Neonatal Unit, Hospital Universitari Sant Joan de Déu, Barcelona, Spain; 4 Department of Paediatrics, Hospital Universitari Sant Joan de Reus, Universitat Rovira i Virgili, Reus (Tarragona), Spain

## Abstract

**Background:**

The notion of Palliative Care (PC) in neonatal and perinatal medicine has largely developed in recent decades. Our aim was to systematically review the literature on this topic, summarise the evolution of care and, based on the available data, suggest a current standard for this type of care.

**Methods:**

Data sources included Medline, the Cochrane Library, CINAHL, and the bibliographies of the papers retrieved. Articles focusing on neonatal/perinatal hospices or PC were included. A qualitative analysis of the content was performed, and data on the lead author, country, year, type of article or design, and direct and indirect subjects were obtained.

**Results:**

Among the 1558 articles retrieved, we did not find a single quantitative empirical study. To study the evolution of the model of care, we ultimately included 101 studies, most of which were from the USA. Fifty of these were comments/reflections, and only 30 were classifiable as clinical studies (half of these were case reports). The analysis revealed a gradual conceptual evolution of the model, which includes the notions of family-centered care, comprehensive care (including bereavement) and early and integrative care (also including the antenatal period). A subset of 27 articles that made special mention of antenatal aspects showed a similar distribution. In this subset, the results of the four descriptive clinical studies showed that, in the context of specific programmes, a significant number of couples (between 37 and 87%) opted for PC and to continue with the pregnancy when the foetus has been diagnosed with a lethal illness.

**Conclusions:**

Despite the interest that PC has aroused in perinatal medicine, there are no evidence-based empirical studies to indicate the best model of care for this clinical setting. The very notion of PC has evolved to encompass perinatal PC, which includes, among other things, the idea of comprehensive care, and early and integrative care initiated antenatally.

## Background

The modern concept of palliative care (PC) has been gaining momentum in recent decades, especially since the 1960s, in response to a realisation that end-of-life issues for seriously ill patients have been inadequately addressed with traditional approaches [1]. The focus on adult PC has reach such relevance that it has become a global public health priority [2].

Although in a slower fashion the concept of PC has been gradually incorporated into neonatology. Only recently it has been accepted that pain and discomfort can affect newborns, whatever their gestational age, and even foetuses [3,4], despite the fact that attention was drawn to this issue already many years ago [5-8]. Likewise, the experience gained in the development of hospices, once again initiated for adults [9] and subsequently adapted to paediatrics and neonatology [10,11], has provided insights towards the PC model applicable to perinatal medicine. The variety of PC approaches has introduced complexity and depth to the concept of PC in perinatal care, which makes necessary some degree of standardization.

Therefore, the objectives of this study were: first, to systematically review the clinical literature on Neonatal Palliative Care (NPC) and Perinatal Palliative Care (PPC) to determine if there is a best model of care; second, to summarise the evolution of the main traits of PPC; and lastly, to identify the most relevant features of PPC currently offered around the world.

## Methods

### Criteria for including studies in this review

We aimed to include clinical trials in which an experimental model of care was compared to another model of care. We planned to include randomised controlled trials (RCTs), cluster RCTs and quasi-RCTs, and decided that if no RCTs and quasi-RCTs were available, then we would include controlled before-and-after studies. In the event that no experimental studies would fulfil these criteria, articles that met the remaining criteria would be classified and examined, regardless of the study design in order to perform a qualitative synthesis of them.

Participants in the included studies were to be foetus, neonates and families who received care guided by a PC model. We did not place any restrictions on diagnosis or clinical setting (e.g. hospital, home or nursing home). We considered measures of the following types of outcomes: physical, psychological, quality of life, and any adverse effects. We excluded studies that focused only on a very specific aspect of the care, such as treatment of pain or ethical decision-making, not specifically in the context of PC.

### Search methods to identify studies

We searched the Cochrane Library, MEDLINE (through PubMed) and CINAHL. The search strategy was developed to comprise searches both for keywords and medical subject headings under existing database organisational schemes. The strategy for MEDLINE (PubMed) is presented in Table 1. No language restriction was considered. The timeframe covered for the databases used in the search was from their inception to May 2010. We searched the reference lists of all relevant reviews or other studies, and scanned paper issues of the journals relevant to our topic.

**Table 1:** Bibliographic search strategy

	Terms used in PubMed (2/5/2010)	Number of hits
#1	*"infant, newborn"[MeSH Term]*	433377

#2	*"perinatal care"[MeSH Term]*	1842

#3	*"perinatology"[MeSH Term]*	1512

#4	Foetus [Title/Abstract]	45084

#5	Fetal [Title/Abstract]	161517

#6	*"palliative-care"[MeSH term]*	32149

#7	Hospice [Title/Abstract]	6011

#8	(#1 OR #2 OR #3 OR #4 OR #5) AND (#6 OR #7)	**1299**

### Selection of studies

**Figure 1:**
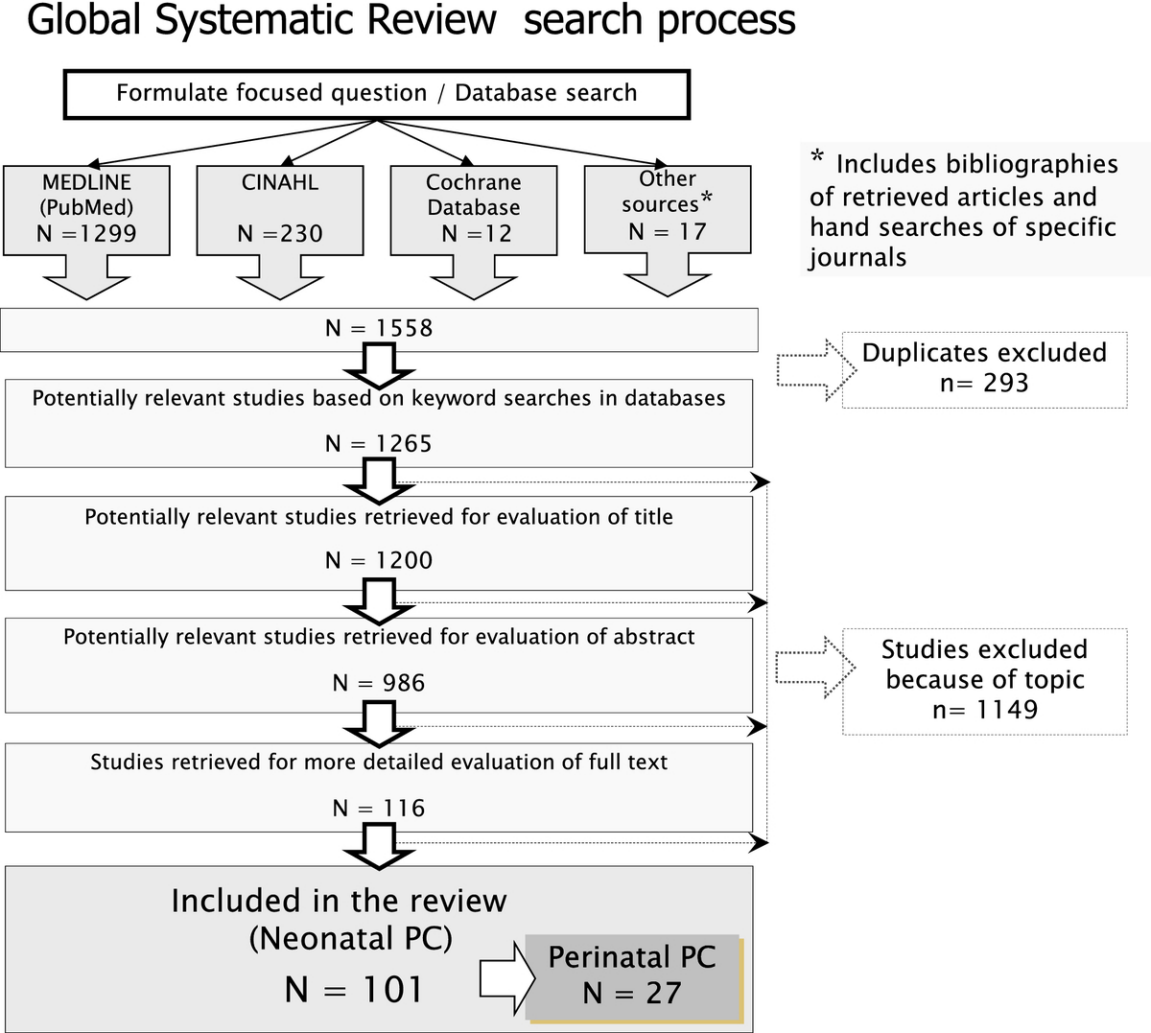
**Flowchart of search results**.

Two review authors (AB, AM) pre-screened all search results (titles and abstracts) for possible inclusion, and those selected by one or both authors were subject to full-text assessment. Disagreements over whether a study met the inclusion criteria were planned to settle through joint discussion among the members of the research team; although there were no discrepancies The search process that we followed is illustrated in Figure 1.

### Data collection and analysis

We first drew up a classification to catalogue the articles found. The categories established were: 1) prospective quantitative clinical studies (including cohort studies and controlled trials); 2) qualitative clinical studies; 3) case-control studies; 4) cross-sectional studies (including surveys on attitudes towards hospices or related issues); 5) case reports and case-series; 6) articles designing, implementing or describing a palliative care programme; 7) literature reviews (discerning narrative reviews from systematic reviews & meta-analyses); 8) guidelines (including evidence-based clinical guidelines, clinical protocols and consensus); 9) comments/reflections; and 10) cost-effectiveness analysis. Those articles that could have been placed in multiple categories were classified into the most appropriate one by consensus among the members of the research group. We agreed that new categories could emerge or that already classified articles could be subject to reclassification.

In addition to performing a qualitative analysis of the texts, the following data from each classified article were recorded on predetermined spreadsheet: lead author and country; year; type of article or design; main topic; direct subjects and number if appropriate; indirect subjects and number if appropriate; and job or position of the authors. A secondary analysis was planned for those articles that envisaged initiating early or prenatal PC, as well as standard care (*i.e*. perinatal palliative care [PPC]).

## Results

In total, 1558 titles and abstracts were retrieved and assessed; there was not a single experimental study that fulfilled the eligibility criteria. Therefore, we classified and examine all the articles that met the remaining criteria, regardless of the study design. The articles were classified according to type of article or design as follows: comments or reflections 50, clinical studies 30 (case reports 15, quantitative series 10, and qualitative series 5), guidelines/clinical practice proposals 11, papers designing/describing a PC programme 5, and reviews 5. According to their place of origin 64 articles were from the USA (mainly from California 11, and Wisconsin 7); 25 from Europe (mainly from the UK 11, France 4, and Germany 3) and the rest were from Australia and New Zealand 6; Canada 4; Hong Kong 1 and Saudi Arabia 1. No quantitative empirical research studies were found, whether experimental (e.g. randomised controlled trials) or observational (cohort, or case-control studies).

**Figure 2:**
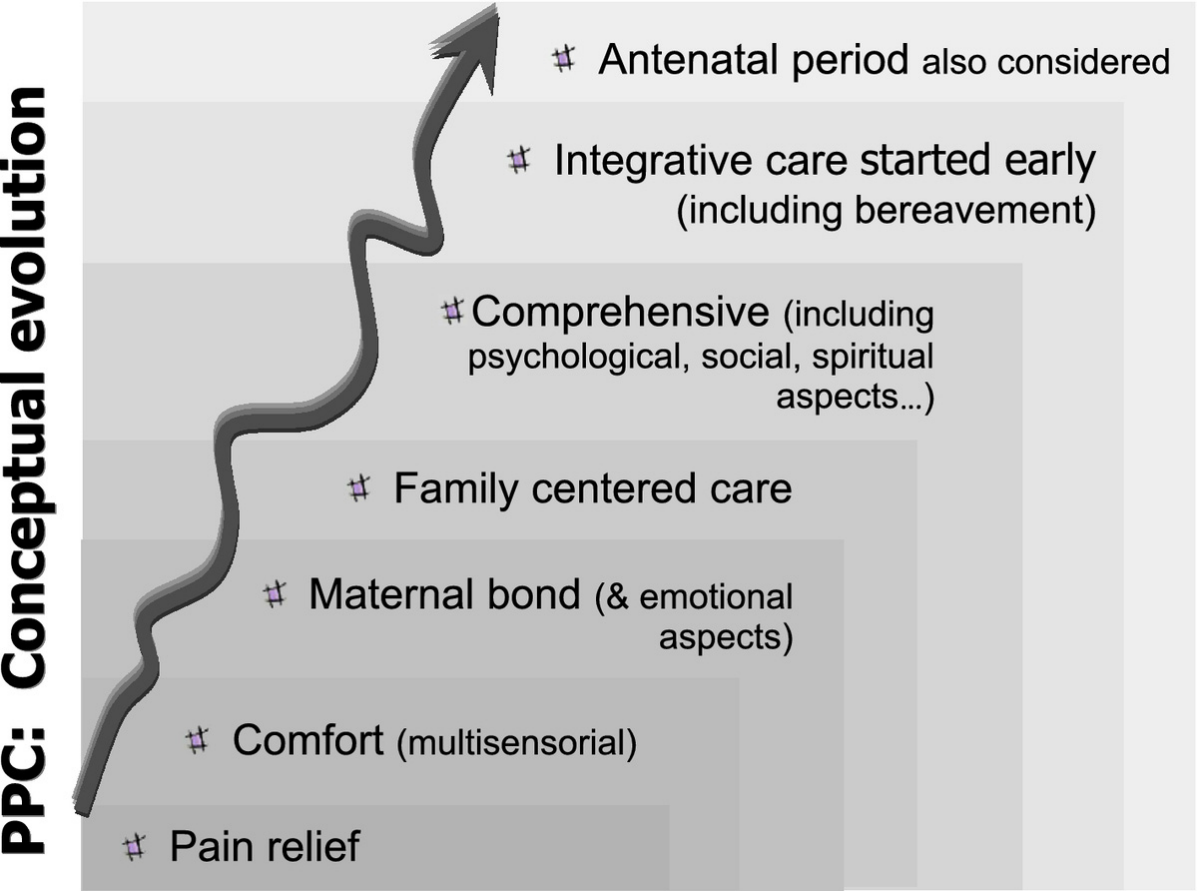
**Conceptual evolution of Perinatal Palliative Care (PPC)**.

Qualitative analysis of the content of the articles showed that the concept of PC has developed gradually; over time, there is a progression in the characterization of the care and consideration of issues that had not been initially addressed. Although the development is not perfectly defined--the various aspects of PC are inter-related and overlap--it can be summarised as follows: a) pain relief; b) comfort (multisensorial context); c) maternal bonding (and other emotional aspects); d) family-centered care; e) comprehensiveness (including psychological, social and spiritual aspects); f) early start and integrative care (including bereavement); g) antenatal period (see Figure 2).

The 27 articles that were considered to be about PPC (those that made explicit mention of preparing or initiating the programme before birth) were subject to a secondary, manual analysis. The distribution of this subgroup by type of article or design (see Table 2) gave percentages that were very similar to those observed in the whole sample. There were eight clinical studies (30%), four of which were quantitative series, three case reports and one a qualitative study. Five (18%) were classified as guidelines/clinical practice proposals and one as designing/describing a PPC programme. As in the whole sample, the highest percentage was for comments/reflections with 13 articles (48%). As far as the country of origin was concerned, the distribution was also similar to that of the sample as a whole: seventeen articles (63%) were from the USA, followed by seven (26%) from Europe (the United Kingdom had the most), and three from Canada. In this subgroup, only four clinical studies were found to show the quantitative results of their programmes. Table 3 shows an extract of the characteristics of these studies and their results.

**Table 2:** PPC: classification of articles by design or main focus (N = 27)

Kind of study	N	Reference
**Comments/reflections**	**13**	Calhoun 1997 [12], Hoeldtke 2001 [13], Milstein 2005 [14], Bhatia 2006 [15], Buus-Frank 2006 [16], Pearce 2006 [17], Sumner 2006 [18], Munson 2007 [19], Pignotti 2007 [20], Roy 2007 [21], Williams 2008 [22], Bétrémieux 2009 [23], Payot 2009 [24]

**Clinical studies**	**8**	

Case reports	3	Watkins 1989 [25], Flower 1992 [26], Nuutila 2008 [27]

Quantitative (series)	4	Calhoun 2003 [1], D'Almeida 2006 [28], Breeze 2007 [29], Leuthner 2007 [30]

Qualitative (series)	1	Chitty 1996 [31]

**Guidelines/clinical practice proposals**	**5**	Craig 2003 [32], Leuthner 2004 [33], Leuthner 2004 (b) [34], Ramer-Chrastek 2005 [35], Howard 2006 [36]

**Designing/describing a PC programme**	**1**	Catlin 2002 [37]

**Table 3:** Summary of clinical studies that published results of perinatal programmes (including antenatal care)

Reference	Design	Years	Eligible Patients	Pregnancy continued	Antenatal death	City-Country	Centre
**Calhoun 2003**	Retro spective	**1995-04**	**31**	**27 (87%)**	**10**	Tacoma (WA, USA)	Madigan Army Medical Centre
						
		**1996-99**				Sacramento (CA, USA)	Travis Air Force Medical Centre

**D'Almeida 2006**	Retro spective	**2000-04**	**28**	**21 (75%)**	**5**	Rockford (IL, USA)	Rockford Memorial Hospital

**Breeze 2007**	Pro spective	**2001-05**	**20**	**8 (40%)**	**2**	Cambridge (UK)	Addenbrooke's Hospital

**Leuthner 2007**	Retro spective	**2000-07**	**185**	**68 (37%)**	No data	Wauwatosa (WI, USA)	Froedtert H. and Children's H. Wisconsin

## Discussion

The field of neonatal and perinatal medicine has been affected by the general interest shown in PC. The first references in the literature referring to the concept as such date to 1982 [11,38], although its origins actually go back to the reaction to therapeutic obstination with premature births at the limit of viability in the early 1970s [39]. However, it should be pointed out that very few clinical studies can be found that can provide empirical data on PC in the perinatal setting. About half of the 101 articles identified were comments/reflections, and less than a third could be considered to be clinical contributions or studies, of which half were simply case reports. Of the clinical contributions, five were classified as primarily qualitative studies [31,40-43], although in some other articles qualitative techniques were used. It was finally decided to classify these five studies, despite the fact that their main aim was not to study PC but to analyse the decision-making process of couples faced with the diagnosis of an unhealthy or non-viable foetus. In contrast, three other qualitative studies by Swanson-Kauffman that focused on the experience of miscarriage and the caring needs of women who miscarry were not included in the classification.

Interest in neonatal/perinatal PC seems to be greater in the USA (followed by Europe) than in other parts of the world, although this distribution may reflect a publishing bias that is influenced by the databases consulted and the lack of clinical literature from some parts of the world, such as Africa. However, it should be borne in mind that sociological and clinical practice differences may imply underlying different meanings regarding PC and end of life issues.

This study has certain limitations, the greatest of which is a lack of evidence-based empirical studies to identify the best model for perinatal PC. Much of the information has not been published in the traditional literature; rather, it is compiled in reports and protocols of clinical practice, which are not immediately available (except a few which are available online [44]) and could introduce some level of publication bias. Given the nature of the articles and the lack of quantitative results, we did a consensus analysis which would allow us to summarize the evolution of PC.

The qualitative evaluation of these articles seems to show an evolution on PPC over time that includes some of the aspects that have also been developed in newborn care. In addition, the care provided has also been enriched by input from palliative care units for adults and children. So the initial care provided for the more physical aspects such as pain relief and comfort (in a multisensorial context) is immediately supplemented with the importance of maternal bonding and other emotional aspects [45]. In this regard, the hospice model, as the precursor/pioneer of PC, has made a considerable contribution. Hospices emerged as a result of the work by Saunders with adults in the 1960s [9] and were soon advocated for children by Saunders herself [10] and then adapted for neonates by Whitfield [11]. Experience has also shown that general care designed not only to minimise pain in neonates but also to make them more comfortable, promote individualised developmental care [6] and facilitate bonding with the mother can also be of great relevance [7,8]. The importance of family participation in the NICU, which found expression in the concept of "family-centered care" in the 1960s and 1970s [7,46] also could have some influence on neonatal PC [1,19,33,36,37,47]. Although PC emerged in close combination with the NICUs [47], to encourage incorporation of the process in the family environment, the possibility of PC taking place in the home (at least on a temporary basis) was considered [32,48]. This option, however, would depend heavily on the professional support that could be provided and the changing circumstances of the patient and the family [45].

Recently, attention has been drawn to the need for "integrative care" [14]. Using this term, Milstein highlights the importance of introducing healing and palliation (when indicated) alongside curative measures as soon as any diagnosis, especially a critical one, is made as an integrative paradigm of care. He also points out that because loss can be experienced in many conditions, even in the absence of death, bereavement is represented as an on-going, continual process throughout a disease process.

In recent years, particular emphasis has been put on the importance of initiating PC early, even antenatally [1,13,33,37]. Three general areas of implementation have been described [49]: foetus/neonates with lethal congenital anomalies, neonates that are previable or at the limits of viability, and neonates that do not respond to aggressive medical management.

An excellent synthesis of the design and implementation of a programme of this sort [11,13,37,50] can be found in the document drawn up by the British Association of Perinatal Medicine, coordinated by Murdoch and entitled "Framework for clinical practice in perinatal medicine". It divides PC planning into eight stages: a) eligibility of foetus or baby for palliative care; b) family care (including psychological support, creating memories, support of spiritual/personal belief and social support); c) communication and documentation; and d) flexible parallel care planning. The next four stages represent points of care transition: e) pre-birth care; f) transition from active postnatal care to supportive care; g) end-of-life care; and h) post end-of-life care [51].

### Early and/or antenatal palliative care

Initiating early PC in adult cancer patients has recently shown benefits not only in terms of quality of life but also in improving expected outcomes and even survival [52]. In perinatal care, all this does not necessarily justify early initiation, which in this case would involve preparing/initiating the programme antenatally. Recently, however, some have called attention to the importance of this early integrative care [1,13,14]. Early initiation (starting from diagnosis) may make a great deal of sense to those parents who must cope with a tragic prenatal diagnosis. Although many institutions are able to provide this sort of care, in some cases it has been explicitly organised in the form of perinatal hospices or PPC programs [1,13]. They have given special attention not only to the curative needs of the fetus and the mother (e.g. clinical complications in the pregnancy) but also to psychological, spiritual and social needs of the whole family. All these actions provided in the right time with coordination amongst all health professional implicated. A secondary analysis of the bibliography identified a subset of 27 articles that make explicit mention of this concept. The geographical distribution and the topics covered were very similar to those of the whole sample of articles. Once again, it is noteworthy that most of the articles can be classified as comments/reflections and that only 30% (8 articles) could be considered to be clinical studies. Of these, three were case reports, one was a qualitative study [31] and four are the results of initiating programmes of this sort [1,28-30]. These programmes were implemented in five different centres, four of which were in different states in the USA and one of which, from the United Kingdom. According to the data provided (summarised in Table 3) and in the context of the PPC programme, the percentage of couples who decided to continue with the pregnancy despite an ominous prenatal diagnosis ranged from approximately 40% [30] to 85% [1]. These programmes involved 124 pregnancies and there was no maternal morbidity. Those parents who chose this model of care gave positive feedback about their decision and the care provided. The sample probably presents biases, because the parents' choice of centre was surely influenced by their *a priori *convictions. Nevertheless, the data highlights that this model of PPC is viable and that many families request it and are grateful for it. Besides the quality of clinical care given to the foetus/neonate, this fact might suggest that, by choosing PPC, parents do not have to cope with the consequences of voluntarily terminating the pregnancy [31,53]. Parents and relatives would be able to cope better with bereavement because they might prepare for the death of the neonate and, even accompany the baby to his/her natural end [29,54]. In any case, when trying to make a decision after a problem with the foetus has been identified [42], parents and patients should have all the appropriate information and support about possible treatments and palliative care.

## Conclusion

**Figure 3:**
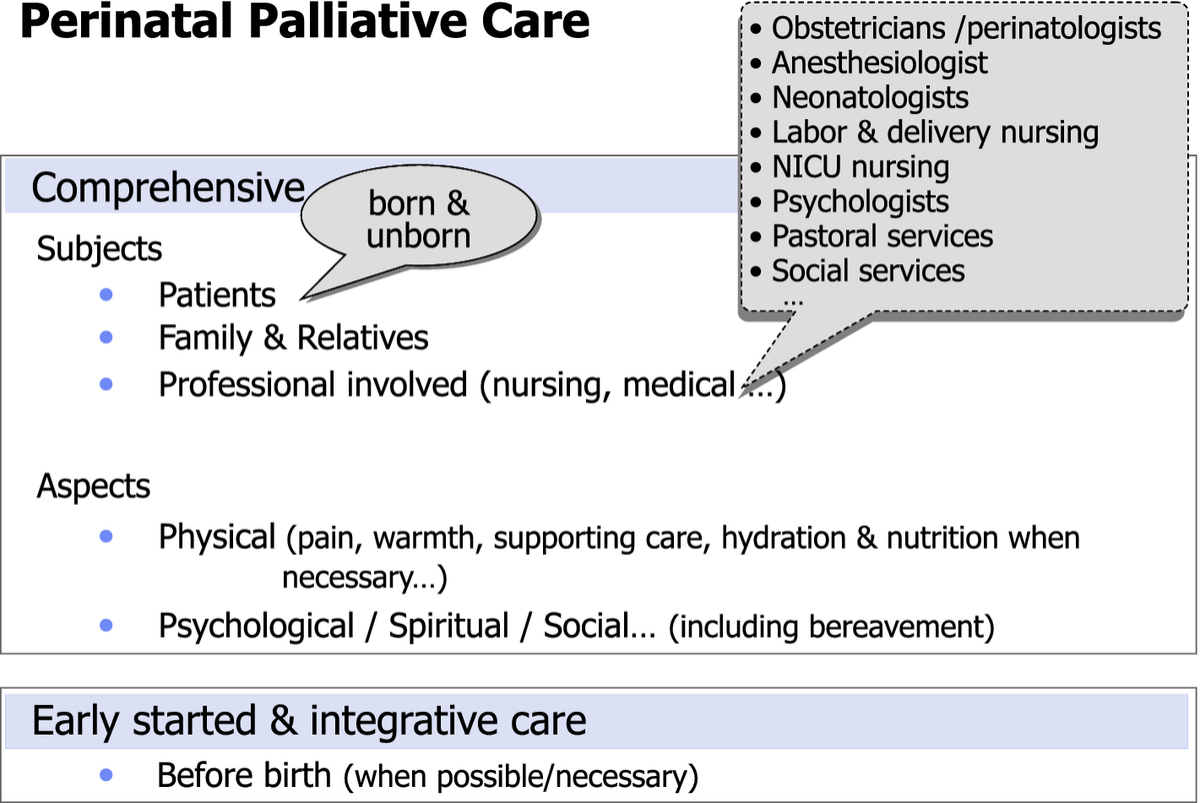
**Suggested standard of excellence for Perinatal Palliative Care (PPC)**.

In summary, in light of the significance and complexity of PC, it seems desirable for obstetric and neonatal units to have available an active and efficient PPC programme. The current literature suggests that PC programmes in perinatal medicine may be comprehensive, initiated early and be integrative (see Figure 3). This comprehensiveness should take into account not only all the people involved (the patient as the centre of the process, including the family and the professionals) but also the aspects to be treated (physical, psychological, spiritual and social, including bereavement). Furthermore, when necessary, palliative care should be planned and initiated before birth. These may be the initial steps towards a model which needs to be further developed.

## Abbreviations

PC: Palliative care; NPC: Neonatal palliative care; NICU: Neonatal intensive care unit; PPC: Perinatal palliative care.

## Competing interests

The authors declare that they have no competing interests.

Some preliminary results of this study were presented at the Global Congress of Maternal and Infant Health on 22-26September, 2010 in Barcelona, Spain.

## Authors' contributions

AB (neonatologist) conceived the study, developed the search strategy, contributed to data collection, abstraction and interpretation and drafted the first manuscript. AMA (neonatologist) and DOE (pediatrician in training) contributed to study design, data collection, abstraction and interpretation and provided critical revisions to the manuscript. JE (pediatrician) and JA (pediatrician and bioethicist) participated in the development of the analytical framework for the study and contributed to the writing of the manuscript. All authors approved the final version of the manuscript.

### Ethical approval

The protocol for this study was discussed with the ethics committees of the authors' hospitals, but formal review was not required.

## Pre-publication history

The pre-publication history for this paper can be accessed here:

http://www.biomedcentral.com/1471-2431/12/25/prepub

## References

[B1] Calhoun BC Napolitano P Terry M Bussey C Hoeldtke NJ Perinatal hospice. Comprehensive care for the family of the fetus with a lethal condition J Reprod Med 2003 48 343 348 12815907

[B2] World Health Organization 2004 http://www.who.int/cancer/palliative/definition/en/accessed October 7, 2010

[B3] American Academy of Pediatrics Committee on Fetus and Newborn Prevention and management of pain in the neonate: an update Pediatrics 2006 118 2231 2241 17079598 10.1542/peds.2006-2277

[B4] Tighe M Fetuses can feel pain BMJ 2006 332 7548 1036 16644849 10.1136/bmj.332.7548.1036 PMC1450060

[B5] Valman HB Pearson JF What the fetus feels BMJ 1980 280 233 234 10.1136/bmj.280.6209.233 7427089 PMC1600041

[B6] Als H Towards a synactive theory of development: promise for the assessment of infant individuality Infant Ment Health J 1982 3 229 243 10.1002/1097-0355(198224)3:4<229::AID-IMHJ2280030405>3.0.CO;2-H

[B7] Warrick LH Family-Centered Care Am J Nursing 1971 1 2134 2138 5209330

[B8] Hostler SL Family-centered care Pediatr Clin North Am 1991 38 1545 1560 1945556 10.1016/s0031-3955(16)38236-0

[B9] Saunders C Terminal patient care Geriatrics 1966 21 70 74 4162818

[B10] Saunders C The management of fatal illness in childhood Proc R Soc Med 1969 62 550 553 5802726 10.1177/003591576906200609 PMC1811078

[B11] Whitfield JM Siegel RE Glicken AD Harmon RJ Powers LK Goldson EJ The application of hospice concepts to neonatal care Am J Dis Child 1982 136 421 424 6177238 10.1001/archpedi.1982.03970410039009

[B12] Calhoun BC Reitman JS Hoeldtke NJ Perinatal hospice: a response to partial birth abortion for infants with congenital defects Issues Law Med 1997 13 2 125 143 9361478

[B13] Hoeldtke NJ Calhoun BC Perinatal hospice Am J Obstet Gynecol 2001 185 525 529 10.1067/mob.2001.116093 11568772

[B14] Milstein J A paradigm of integrative care: healing with curing throughout life, "being with" and "doing to" J Perinatol 2005 25 563 568 10.1038/sj.jp.7211358 16034476

[B15] Bhatia J Palliative care in the fetus and newborn J Perinatol 2006 1 24-6 discussion S31-3 10.1038/sj.jp.7211468 16625220

[B16] Buus-Frank ME Sometimes a time to be born is also a time to die Adv Neonatal Care 2006 6 1 1 3 10.1016/j.adnc.2005.12.001 16458245

[B17] Pearce EW Lewis P A hospice for the pre-born and newborn. A Kansas city facility provides care for babies with severe birth defects and for their families, too Health Prog 2006 87 5 56 61 16986472

[B18] Summer LH Kavanaugh K Moro T Extending palliative care into pregnancy and the immediate newborn period J Perinat Neonatal Nurs 2006 20 1 113e6 16508478 10.1097/00005237-200601000-00032

[B19] Munson D Leuthner SR Palliative care for the family carrying a fetus with a life-limiting diagnosis Pediatr Clin North Am 2007 54 787 798 17933623 10.1016/j.pcl.2007.06.006

[B20] Pignotti MS The Italian law on termination of pregnancy (194/1978). Should it be revised? The palliative care option Recenti Prog Med 2007 98 12 607 610 18369034

[B21] Roy DJ When newborn babies have to die... perinatal palliative care? J Palliat Care 2007 23 2 67 68 17853841

[B22] Williams C Munson D Zupancic J Kirpalani H Supporting bereaved parents: practical steps in providing compassionate perinatal and neonatal end-of-life care A North American perspective Semin Fetal Neonatal Med 2008 13 5 335 340 10.1016/j.siny.2008.03.005 18472317

[B23] Bétrémieux P Palliative care of the newborn, how is it possible? Arch Pediatr 2009 16 6 603 605 10.1016/S0929-693X(09)74083-7 19541101

[B24] Payot A Prenatal palliative care: a challenge of consistency between prenatal and postnatal care Arch Pediatr 2009 16 6 597 599 10.1016/S0929-693X(09)74081-3 19541099

[B25] Watkins D An alternative to termination of pregnancy Practitioner 1989 233 1472 990 992 2594680

[B26] Flower BL Dare we not care? Conflict in the newborn nursery J Christ Nurs 1992 9 2 4 6 1564638 10.1097/00005217-199209020-00002

[B27] Nuutila M Saisto T Prenatal diagnosis of vein of Galen malformation: a multidisciplinary challenge Am J Perinatol 2008 25 4 225 227 10.1055/s-2008-1066877 18548395

[B28] D'Almeida M Hume RF Lathrop A Njoku A Calhoun BC Perinatal Hospice: Family-Centered Care of the Fetus with a Lethal Condition J Am Physicians and Surgeons 2006 11 52 55

[B29] Breeze AC Lees CC Kumar A Missfelder-Lobos HH Murdoch EM Palliative care for prenatally diagnosed lethal fetal abnormality Arch Dis Child Fetal Neonatal Ed 2007 92 F56 F58 10.1136/adc.2005.092122 16705007 PMC2675307

[B30] Leuthner S Jones EL Fetal Concerns Program: a model for perinatal palliative care MCN Am J Matern Child Nurs 2007 32 272 278 10.1097/01.NMC.0000287996.90307.c6 17728587

[B31] Chitty LS Barnes CA Berry C Continuing with pregnancy after a diagnosis of lethal abnormality: experience of five couples and recommendations for management BMJ 1996 313 478 480 10.1136/bmj.313.7055.478 8776321 PMC2351873

[B32] Craig F Goldman A Home management of the dying NICU patient Semin Neonatol 2003 8 177 183 10.1016/S1084-2756(02)00223-3 15001154

[B33] Leuthner SR Palliative care of the infant with lethal anomalies Pediatr Clin North Am 2004 51 747 759 10.1016/j.pcl.2004.01.006 15157596

[B34] Leuthner SR Fetal palliative care Clin Perinatol 2004 31 3 649 665 10.1016/j.clp.2004.04.018 15325543

[B35] Ramer-Chrastek J Thygeson MV A perinatal hospice for an unborn child with a life-limiting condition Int J Palliat Nurs 2005 11 6 274 276 16010223 10.12968/ijpn.2005.11.6.18294

[B36] Howard ED Family-centered care in the context of fetal abnormality J Perinat Neonatal Nurs 2006 20 237 242 16915056 10.1097/00005237-200607000-00011

[B37] Catlin A Carter B Creation of a neonatal end-of-life palliative care protocol J Perinatol 2002 22 184 195 10.1038/sj.jp.7210687 11948380

[B38] Silverman WA A hospice setting for humane neonatal death Pediatrics 1982 69 239 7058104

[B39] Duff RS Campbell AGM Moral and ethical dilemmas In special care nurseries N Engl J Med 1973 289 890 894 10.1056/NEJM197310252891705 4729120

[B40] Locock L Crawford J Crawford J The parents' journey: continuing a pregnancy after a diagnosis of Patau's syndrome BMJ 2005 331 1186 1189 10.1136/bmj.331.7526.1186 16293841 PMC1285101

[B41] Redlinger-Grosse K Bernhardt BA Berg K Muenke M Biesecker BB The decision to continue: the experiences and needs of parents who receive a prenatal diagnosis of holoprosencephaly Am J Med Genet 2002 112 369 378 10.1002/ajmg.10657 12376939

[B42] Sandelowski M Jones LC Healing fictions': stories of choosing in the aftermath of the detection of fetal anomalies Soc Sci Med 1996 42 353 361 10.1016/0277-9536(95)00102-6 8658230

[B43] Sandelowski M Barroso J The travesty of choosing after positive prenatal diagnosis J Obstet Gynecol Neonatal Nurs 2005 34 307 318 10.1177/0884217505276291 15890829

[B44] Perinatal Hospice and Palliative Care http://www.perinatalhospice.org/accessed Novembre 6, 2010

[B45] Butler NC The NICU culture versus the hospice culture: can they mix? Neonatal Netw 1986 5 35 42 2430166

[B46] Darnell B We give our preemies family-centered care RN 1966 29 57 61 5176143

[B47] Walden M Sudia-Robinson T Carrier CT Comfort Care for Infants in the Neonatal Intensive Care Unit at End of Life Newborn and Infant Nursing Reviews 2001 1 97 105 10.1053/nbin.2001.25436

[B48] Cavaliere T Should neonatal palliative care take place at home, rather than the hospital? Pro MCN Am J Matern Child Nurs 2007 32 270 10.1097/01.NMC.0000287994.13179.43 17728585

[B49] Kaempf JW Tomlinson MW Campbell B Ferguson L Stewart VT Counseling pregnant women who may deliver extremely premature infants: medical care guidelines, family choices, and neonatal outcomes Pediatrics 2009 123 1509 1515 10.1542/peds.2008-2215 19482761

[B50] Carter BS Bhatia J Comfort/palliative care guidelines for neonatal practice: development and implementation in an academic medical center J Perinatol 2001 21 279 283 10.1038/sj.jp.7210582 11536019

[B51] British Association of Perinatal Medicine 2009 http://www.bapm.org/publications/documents/guidelines/Palliative_Care_Report_final_%20Aug10.pdf viewed December 21 2010

[B52] Temel JS Greer JA Muzikansky A Gallagher ER Admane S Jackson VA Dahlin CM Blinderman CD Jacobsen J Pirl WF Billings JA Lynch TJ Early palliative care for patients with metastatic non-small-cell lung cancer N Engl J Med 2010 363 733 742 10.1056/NEJMoa1000678 20818875

[B53] Anonymous Women should be offered post-abortion psychological care Lancet 2008 372 602 18722847 10.1016/S0140-6736(08)61251-9

[B54] Lewis E Mourning by the family after a stillbirth or neonatal death Arch Dis Child 1979 54 303 306 10.1136/adc.54.4.303 572201 PMC1545303

